# An enormous sulfur isotope excursion indicates marine anoxia during the end-Triassic mass extinction

**DOI:** 10.1126/sciadv.abb6704

**Published:** 2020-09-09

**Authors:** Tianchen He, Jacopo Dal Corso, Robert J. Newton, Paul B. Wignall, Benjamin J. W. Mills, Simona Todaro, Pietro Di Stefano, Emily C. Turner, Robert A. Jamieson, Vincenzo Randazzo, Manuel Rigo, Rosemary E. Jones, Alexander M. Dunhill

**Affiliations:** 1 School of Earth and Environment, University of Leeds, Leeds, UK.; 2 State Key Laboratory of Biogeology and Environmental Geology, School of Earth Sciences, China University of Geosciences, Wuhan, China.; 3 Department of Earth and Marine Sciences, University of Palermo, Palermo, Italy.; 4 Department of Geosciences, University of Padova, Padova, Italy.; 5 Department of Earth Sciences, University of Oxford, Oxford, UK.

## Abstract

The role of ocean anoxia as a cause of the end-Triassic marine mass extinction is widely debated. Here, we present carbonate-associated sulfate δ^34^S data from sections spanning the Late Triassic–Early Jurassic transition, which document synchronous large positive excursions on a global scale occurring in ~50 thousand years. Biogeochemical modeling demonstrates that this S isotope perturbation is best explained by a fivefold increase in global pyrite burial, consistent with large-scale development of marine anoxia on the Panthalassa margin and northwest European shelf. This pyrite burial event coincides with the loss of Triassic taxa seen in the studied sections. Modeling results also indicate that the pre-event ocean sulfate concentration was low (<1 millimolar), a common feature of many Phanerozoic deoxygenation events. We propose that sulfate scarcity preconditions oceans for the development of anoxia during rapid warming events by increasing the benthic methane flux and the resulting bottom-water oxygen demand.

## Teaser

Low ocean sulfate levels at the end-Triassic mass extinction linked to rapid development of marine anoxia.

## INTRODUCTION

The end-Triassic mass extinction (ETME) is one of the largest known biological crises of the Phanerozoic and is regarded as one of the “Big Five” (*1*). This extinction has been linked with voluminous volcanism during the emplacement of Central Atlantic magmatic province (CAMP) and its associated environmental effects (*2*). These effects include global warming and ocean anoxia. Existing evidence suggests that basinal marine anoxia was widespread on the northern Panthalassan margin of Pangaea and that intense shelf euxinia also became widespread in the latest Triassic–earliest Jurassic of Western Europe, but some of these conditions developed, some ~150 thousand years (ka) after the onset of the ETME (*3*–*6*). Additional findings from seawater δ^238^U in the Lombardy basin of western Tethys suggest an increase in the extent of anoxic deposition through the Triassic-Jurassic boundary (*7*). However, in other oceans, clear evidence for widespread anoxia in the latest Rhaetian that directly coincides with the beginning of ETME has not been recorded, leaving its role as the cause of the marine component of the ETME questionable (*8*).

Carbonate-associated sulfate (CAS) in bulk marine carbonate and biogenic calcite is widely used to reconstruct the primary seawater sulfate S isotope composition during major redox perturbations of the Earth surface system (*9*–*13*). Seawater sulfate δ^34^S is dynamically controlled by variations in the fluxes and isotopic compositions of riverine sulfate sources and marine pyrite burial. The removal of sulfate from the oceans via gypsum precipitation does not impart an isotopic fractionation, but this removal makes the global sulfate reservoir smaller and, therefore, more isotopically susceptible to changes in other fluxes (*14*). The production and burial of pyrite represent a primary redox-sensitive pathway in the marine sulfur cycle, which drives a large offset between the sulfur isotopic composition of the seawater sulfate and sedimentary pyrite pools, and thus may control variations in the S isotope composition of oceanic sulfate (δ^34^S_CAS_) through time. Large and rapid global-scale S isotope perturbations, as well as the small ocean sulfate reservoirs needed to produce them, seem to be a feature of major deoxygenation events of the Phanerozoic (*9*–*13*). Although there is some evidence in the sedimentary pyrite isotope record that suggests the regional development of marine anoxia at the ETME (*5*, *15*, *16*), direct records of changes in the marine sulfate pool and therefore impacts on the global sulfur cycle are undocumented.

**Fig. 1:**
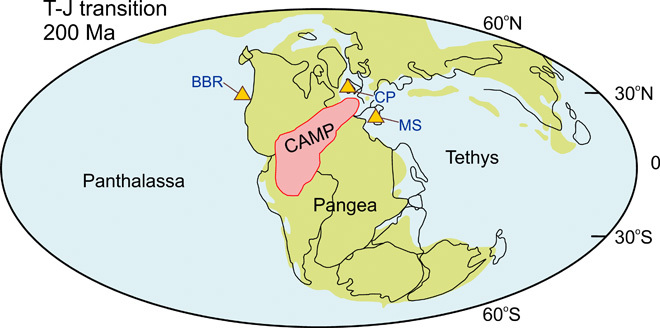
Simplified paleogeographical map for Triassic-Jurassic transition showing localities for all three studied sections. This figure is modified after the work of Luo *et al*. (*16*). T-J, Triassic-Jurassic. Yellow filled triangles indicate the location of studied sections. The paleogeographical location and the extent of CAMP are based on the work of Marzoli *et al*. (*42*).

Here, we report three open marine CAS-δ^34^S_CAS_ profiles from Sicily [Mount Sparagio section (MS)], Northern Ireland [Cloghan Point section (CP)], and British Columbia [Black Bear Ridge section (BBR)] (Fig. 1). These derive from both Tethyan and Panthalassan locations; the first two sections archive well-preserved, shallow-water, peritidal, micritic, and shelly limestones and shell materials (*17*, *18*); and the last section consists of open-shelf, organic-rich, and bivalve-rich marly limestone (*6*, *19*). The sections span the Norian to lower Hettangian and record the major losses of the ETME (*6*, *17*–*19*). Therefore, they provide a window into the possible links between the ecosystem response and marine redox variations in Late Triassic oceans over a broad area (see Materials and Methods and the Supplementary Materials for analytical procedures, stratigraphic correlations, and sample descriptions).

## RESULTS

### Sulfate S isotope trends

**Fig. 2:**
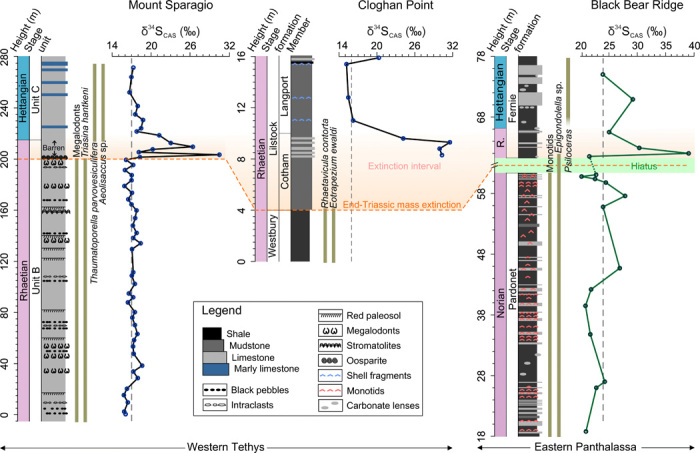
δ^34^S_CAS_ profiles from Late Triassic to Early Jurassic for the three studied sites of the Tethys and Panthalassa oceans. R., Rhaetian. The orange shadowed field indicates the extended extinction interval following the major mass extinction horizon. The light green field indicates a hiatus between Norian and Rhaetian at the BBR. Dark green bars represent the fossil occurrence ranges. See the Supplementary Materials for further stratigraphic details. Vertical dash lines indicate pre- or postexcursion average baseline values.

All δ^34^S_CAS_ profiles from three different localities show similar trends (Fig. 2), although the absolute values vary between the European and North American sections (see the Supplementary Materials for evaluation of diagenesis and data). In all sections, a large positive δ^34^S_CAS_ shift with a magnitude of >10 per mil (‰) is seen in the latest Rhaetian [~201.5 million years (Ma) ago] and coincides precisely with the extinction horizon (Fig. 2). Two consecutive positive δ^34^S_CAS_ excursions are shown at the MS, while only a single spike is seen at the other two sections. At the CP, only the falling limb of the positive excursion was recovered because of the absence of suitable bulk carbonate or shell material below this level. The pre- and postexcursion baseline values for the two Tethyan sections are between 15 and 20‰, which are close to the existing global δ^34^S_CAS_ and evaporite dataset for the Late Triassic (*20*). By contrast, the δ^34^S_CAS_ record at the Panthalassa BBR generally yields more positive baseline values and a slightly larger positive swing (see discussion in the Supplementary Materials). This is likely due to the development of sulfate isotopic and concentration heterogeneity between Tethyan and Panthalassan sites under low sulfate conditions (*9*, *11*). Note also that the positive sulfate δ^34^S_CAS_ excursion at the BBR is mirrored by synchronous positive δ^34^S shifts in sedimentary pyrite at a deeper site (Kennecott Point section) in eastern Panthalassa (*15*), suggesting a coupled behavior in both marine oxidized and reduced sulfur sinks.

### Duration of the S isotope excursion event

We calculated the age model at the most stratigraphically complete Tethyan MS (Fig. 2). The duration of the shift from the baseline value (~16 to 17‰) to the first peak value (~31‰) is estimated to take ~50 ka with the assumption of a constant sedimentation rate and a Rhaetian duration of 4.1 Ma (see the Supplementary Materials for details). This time frame is broadly in agreement with the equally short-lived major phase of the extinction, which was proposed to last for ~40 ka (*21*). Thus, the observed δ^34^S_CAS_-positive excursion event in the latest Triassic appears to represent an extreme and short-lived perturbation when compared to other similar positive S isotope events during, for example, the end-Permian extinction (~100 ka) (*12*), Toarcian oceanic anoxic event (OAE) (~1 Ma) (*9*, *11*), and Cretaceous OAE 2 (~0.5 Ma) (*10*).

## DISCUSSION

### Latest Triassic anoxia, enhanced pyrite burial, and low marine sulfate

The observed positive swing in the S isotope composition of seawater sulfate in the latest Triassic could have been driven by an increase in the net burial of sedimentary pyrite under expanded anoxic/euxinic conditions (*22*). These conditions result in enhanced microbial sulfate reduction (SR), leading to an enhanced pyrite burial flux on the continental shelves and slopes when there is sufficient supply of available iron and organic matter. Because pyrite is depleted in the heavier isotope ^34^S, elevated burial fluxes on a global scale would drive the seawater sulfate δ^34^S to more positive values. The oxidative biotic pathway of the global sulfur cycle may also have the potential to drive seawater sulfate δ^34^S enrichment to some extent via microbial sulfide oxidation by some sulfide-oxidizing microorganisms (*23*). However, the contribution of this oxidative metabolic pathway to the oceanic sulfate pool remains unclear, and there is no obvious mechanism for it to have driven a prolonged positive S isotope excursion in the global seawater sulfate inventory. On a larger scale, it may be possible to drive S isotope variations by altering the weathering rates of continental pyrite and gypsum; here, a geologically sudden increase in seawater δ^34^S might represent a cessation of pyrite weathering and a switch to an isotopically heavy riverine flux.

**Fig. 3:**
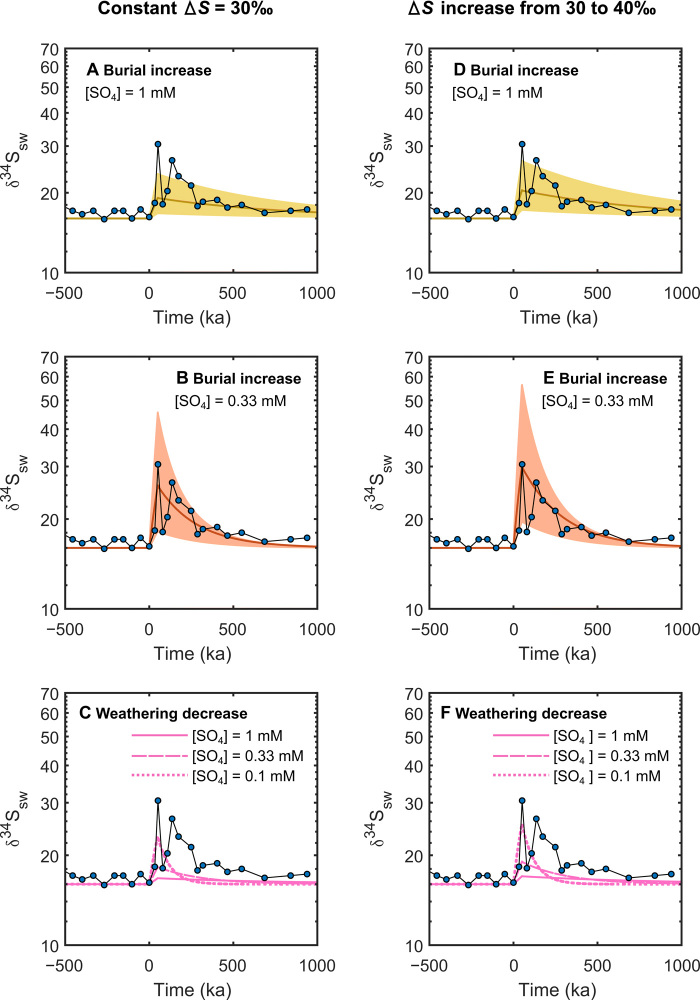
Sulfur cycle box model outputs. (**A** and **B**) Increased in the pyrite burial rate under different values for the starting oceanic sulfate inventory, with tests of 1 mM (A) (yellow) and 0.33 mM (B) (red). For both scenarios, a step increase in pyrite burial is assumed to occur at *t* = 0 over a period of 50 ka, which represents the ETME. Both models assume the same increase in pyrite burial rates, which ranges from 2- to 10-fold to create the shaded area, with the centerline showing a fivefold increase. The best fit to the data occurs for marine sulfate concentration [SO_4_] = 0.33 mM (B). (**C**) Attempts to fit the δ^34^S_CAS_ data by instead reducing the pyrite weathering rate to zero over the same 50-ka time frame. Here, regardless of [SO_4_], the shape of the curve cannot be fit. This is because creating the large excursion this way requires extremely low [SO_4_], and, in these circumstances, the system is quick to regain isotopic stability. (**D** to **F**) Repetition of these experiments with the addition of a change in the enrichment factor Δ*S* between oceanic sulfate and sedimentary pyrite and continuation to produce a better fit when [SO_4_] = 0.33 mM.

To investigate the response of seawater sulfate δ^34^S to the variations of oceanic sulfate inventory and the degree of change in the net pyrite burial flux, we applied a time-dependent sulfur cycle single-box model (*24*). The model assumes that the isotopic composition of the pyrite and gypsum weathering fluxes remain constant, and experiments then alter the pyrite input and output fluxes through either weathering or burial. Full model details are in Materials and Methods and the Supplementary Materials. Figure 3 (A and B) shows the results of increasing the pyrite sulfur burial flux for 50 ka by a factor of between 2 and 10 to simulate black shale deposition, driven by large-scale marine anoxia and increased supply of nutrients from warming-induced continental weathering (*2*). In the model, a substantial increase in pyrite burial by approximately a factor of 5 and a very small marine sulfate reservoir (<1 mM) is required to replicate the magnitude and timing of the δ^34^S_CAS_ shift (Fig. 3B). The version of the model in Fig. 3 (A to C) fixes the isotopic enrichment of buried pyrite at 30‰ more negative than contemporaneous seawater sulfate, but the expansion of euxinia may have increased this enrichment factor; thus, we also experiment with a scenario in which this is increased to 40‰ during the event (Fig. 3, D to F) (*10*). This experiment has a very similar requirement for a large increase in pyrite burial and very low seawater sulfate concentration. Note that it is the size, direction, and duration of change that are the important foundations of our modeling approach. Differences in regional sulfate isotope baselines have no impact on the conclusions from the modeling work, as a similar sized isotope excursion is present in all records. Replicating the change in δ^34^S by reducing pyrite weathering rates while maintaining the same gypsum weathering flux (Fig. 3, C and F) is much more difficult and requires a complete cessation of pyrite weathering and extremely low ocean sulfate (~0.1 mM). Even then, the shape of the excursion is not readily reproducible, as the very low sulfate concentrations mean that the system rapidly recovers from the perturbation.

The maximum marine sulfate concentrations can be independently estimated using the maximum rate of change in δ^34^S_CAS_. The “rate method” model (*13*, *25*) gives an upper estimate for marine sulfate of ~0.2 to 1.1 mM for the interval through the Late Triassic–positive isotope excursion event (Materials and Methods give the model details). The lower end of these maximum estimates is consistent with the calculations inferred from our sulfur cycle box model (Fig. 3). Therefore, the intervals predating and during the positive S isotope excursion event appear to be characterized by a scarcity of oceanic sulfate when compared to a higher fluid inclusion–based estimate of ≥13 mM during the Carnian, although this was ca. 20 Ma earlier (*26*). The development of a low sulfate ocean in the later Triassic was likely caused by substantial evaporite deposition. As shown in global compilations for this interval (*27*), minimum estimates of global halite deposition suggest a 16-fold increase from the Middle to Late Triassic. By contrast, the earlier part of the Triassic experienced a low level of evaporite occurrence following the end-Permian extinction (*27*). Late Triassic evaporites were deposited in newly formed rift basins that developed in an arid climate as Pangaea began to break up (*27*). When examined on a regional scale, for example, evaporite deposition became widespread surrounding the North Atlantic rift (northeastern Grand Banks, Oranian meseta, and Western Europe) during the Late Triassic and subsequently peaked in the Earliest Jurassic (*28*).

### Low sulfate facilitates the rapid expansion of anoxia during warming

**Fig. 4:**
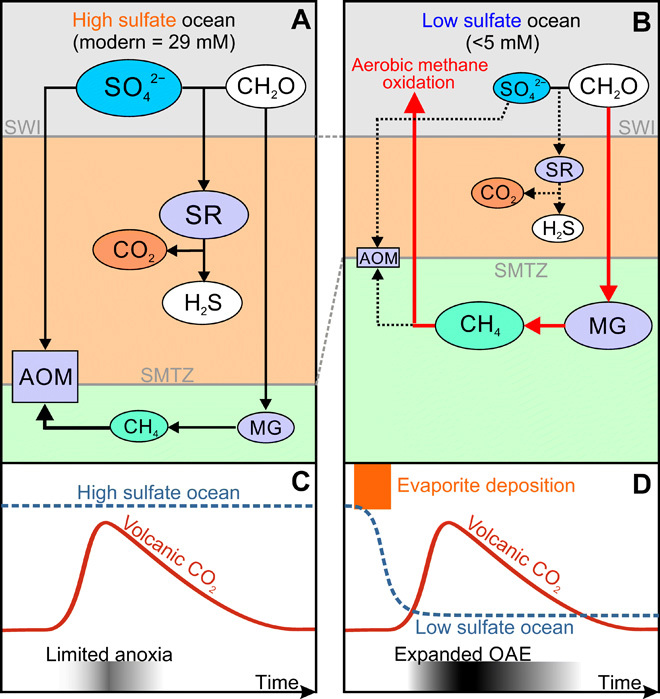
Conceptual model of the methane-oxygen link under high and low sulfate conditions. (**A**) The fate of organic carbon in the modern high sulfate ocean: More organic carbon and methane are oxidized by sulfate with negligible benthic methane flux, which limits water column oxygen demand. (**B**) The effect of enhanced methanogenesis in a low sulfate setting: The proportion of organic carbon available for methane production is increased, sulfate-driven anaerobic AOM oxidation is suppressed, and methane production moves closer to the sediment surface producing a high benthic oxygen demand. Red arrows in (B) indicate acceleration of biogeochemical pathways relative to modern, whereas dotted black arrows indicate retardation. (**C** and **D**) The envisaged oxygen depletion responses of the ocean to the same CO_2_ forcing under high and low sulfate conditions. Sulfate is thought to be removed by evaporite deposition. Marine anoxia is exacerbated by the increased oxygen demand as net seafloor methane fluxes increase during warming. MG, methanogenesis. SMTZ, sulfate-methane transition zone.

Our finding of low marine sulfate concentrations preceding an episode of massive pyrite burial in the latest Triassic adds to an increasing number of studies that link low seawater sulfate with the expansion of anoxic waters in the oceans (table S2) (*9*–*13*). Here, we propose a conceptual model to link these observations. Marine sulfate and organic carbon availability exert a major control over the balance between three microbially mediated biogeochemical pathways in marine sediments (Fig. 4): SR (SO_4_^2−^ + 2CH_2_O → H_2_S + 2 HCO_3_^−^), methanogenesis (CH_3_COO^−^ + H^+^ → CH_4_ + CO_2_ and CO_2_ + 4H_2_ → CH_4_ + 2H_2_O), and the anaerobic oxidation of methane (AOM) (CH_4_ + SO_4_^2−^ → HCO_3_^−^ + HS^−^ + H_2_O). Under high sulfate conditions such as the modern ocean, SR consumes large amounts of organic carbon, while methane is produced deeper in the sediment where sulfate has been depleted. The overlying sulfate-rich pore water fuels AOM and prevents substantial benthic methane escape, therefore limiting bottom-water oxygen consumption (Fig. 4A). In contrast, under conditions of low sulfate availability, the balance of processes oxidizing organic matter in marine sediments shifts in favor of methanogenesis (Fig. 4B), as occurs widely in freshwater sediments (e.g., lakes) (*29*, *30*), where sulfate supply is usually limited. Lower sulfate concentrations bring the sulfate-methane transition zone closer to the sediment-water interface (SWI) and reduce the amount of organic matter consumed by SR, ultimately increasing the organic carbon flux to methanogens and limiting the capacity for anaerobic oxidation of the resulting methane. The organic matter reaching the zone of maximum methanogenesis will also have increased reactivity. The result is a greater flux of methane from the sediment, leading to increased aerobic respiration of methane close to the SWI (Fig. 4B) placing an increased burden on bottom-water O_2_ levels (*12*, *31*, *32*).

In the modern system, around 98% of all buried organic carbon in the ocean is stored in continental margin sediments (*30*). On average, around 20% of the global organic carbon flux (~191 Tmol C year^−1^) to the seafloor is processed via SR, and ~3 to 4% is converted to methane, giving an annual methane flux from seafloor of ~5.7 to 7.6 Tmol CH_4_ year^−1^ (*30*, *33*, *34*). If we assume that a drawdown in oceanic sulfate concentration by ~97% from 29 mM (modern value) to 1 mM will reduce the rate of SR by a similar amount and that the excess organic matter will all be used by methanogens (i.e., they now process ~22 to 23% of the organic carbon), then the methane flux would rise to around ~42 to 44 Tmol CH_4_ year^−1^. This calculation is conservative, since it does not take into account any increase in reactivity of the organic matter reaching the methanogenic zone. Furthermore, suppression of AOM under these low sulfate conditions would make it easier for this methane to reach the water column and consume free O_2_. Making more detailed calculations on the expected impact of low sulfate conditions on water column O_2_ demand requires further modeling, which is beyond the scope of this study, but our calculations demonstrate that there is clear potential for at least a six- or sevenfold elevation in the methane flux at the SWI and a concomitant increase in the global consumption of benthic O_2_. Note that these elevated demands on bottom-water O_2_ exist where sulfate concentrations are low and before any additional drivers from the release of volcanic CO_2_.

Finding evidence for elevated aerobic methane oxidation under low sulfate conditions in the sedimentary record is not simple because the resulting dissolved inorganic carbon (DIC) flux, while large when considered in the context of dissolved oxygen uptake, is small compared to the abundance of ocean DIC, especially when oxidation takes place in the water column as proposed. Isotopically depleted carbonate cements form from pore waters and are a common feature of the sedimentary record and so do not provide definitive evidence. Calcifying organisms living at the SWI are likely to provide the best archive for recording this process, evidence for which has been recognized in high-latitude late Cretaceous bivalves (*31*).

A key feature of our conceptual model is that sulfate poor conditions are established before volcanic perturbation, likely by widespread evaporite deposition (Fig. 4, C and D). Previously, authors have explained the link between the expansion of marine anoxia during large igneous province (LIP)–driven warming and extinction events via the decreased solubility of O_2_ in warmer waters and increased productivity and oxygen demand driven by increased weathering fluxes of nutrients from land and the recycling of phosphorus once euxinic water column conditions are established (*35*). The higher bottom-water oxygen demand of a steady-state Earth system with a small marine sulfate reservoir will predispose the oceans to the rapid expansion of anoxic conditions via these mechanisms. In addition, a low sulfate ocean is likely to impose some additional feedbacks once warming has been initiated: The rate of methanogenesis is highly temperature sensitive (*36*), so methane production will increase with sediment temperature, a situation amplified by the reduced depth to the methanogenic zone under low sulfate conditions. Increased marine organic matter production will increase the delivery of organic matter and its reactivity to the methanogenic zone in sediments, again adding to increased methane fluxes across the SWI and O_2_ consumption from methane oxidation. Pyrite burial will increase as anoxic conditions expand, creating downward pressure on marine sulfate concentrations, although this may be countered by bigger fluxes of weathered sulfate from land. Elevated global marine methane production may also promote methane release to the atmosphere and thereby contribute to warming trends initiated by the large-scale release of volcanic CO_2_, although much of the additional methane production is likely to be oxidized in the water column. These additional feedbacks may explain why the expansion of anoxic conditions is more severe under low sulfate conditions and why not all LIP-driven warming events create widespread oxygen depletion.

### Marine anoxia and mass extinction

Although anoxia may not have developed on the deep ocean floor during the Triassic-Jurassic transition (*8*), other geochemical evidence, in the form of enrichment of redox-sensitive elements (e.g., Mn and Mo) and nitrogen isotope fluctuations, suggests that there was a major intensification of the mid-water oxygen minimum zone (OMZ) in the Panthalassa Ocean at the time (*19*, *37*). Tangible evidence for this is seen where the OMZ impinged on the western margin of the Pangean supercontinent, leading to extensive black shale deposition in Western Canada (*6*, *38*). Euxinia also became extensive in the latest Triassic shelf seas of Western Europe, both during and at the termination of the mass extinction phase (*3*, *5*). Uranium isotope data from marine carbonates provide a possible measure of ocean redox conditions with negative excursions of δ^238^U values signifying enhanced reduction from U(VI) to U(IV) (*7*). Such a signal, seen at the start of the mass extinction, suggests a major increase in the area of anoxic deposition that lasted for ~50 ka (*7*).

Our δ^34^S_CAS_ excursions reveal a similar link between the onset of mass extinction and an anoxia-driven isotopic excursion. The link is most clearly seen in western Tethys (MS) where megalodont bivalves and the foraminifer *Triasina hantkeni* are suddenly lost at the onset of the positive shift (Fig. 2) (*17*). Although there is no direct evidence for anoxia at this peritidal location, some contemporaneous anoxic sedimentary matrices are seen at a neighboring site that was also connected to the western Tethys (*39*). There is a hiatus in the Panthalassan section (BBR), but the extinction level is still recorded. This occurs in the dysoxic strata of the basal Fernie Formation, where the last Rhaetian conodonts disappear, and is coincident with the δ^34^S_CAS_ excursion (Fig. 2) (*6*). The extinction of monotid bivalves at BBR marks an earlier crisis at the end of the Norian, several million years before the end-Triassic event (*6*, *19*). The end-Triassic extinction is also seen at CP, where several bivalve species, including the Rhaetian marker *Rhaetavicula contorta*, disappear at the base of the Cotham Member (Fig. 2). The lack of limestones at this level precludes measurement of δ^34^S_CAS_, but the lowest data point obtained in this section, a short distance above, displays a strongly positive value (Fig. 2). In summary, the major δ^34^S_CAS_ excursion found here is best explained by a major pyrite burial event driven by a large-scale, increase in anoxia in the late Rhaetian. Our age model for the MS section suggests a 50-ka duration for the initial positive shift in δ^34^S_CAS_, a time span in remarkable accord with the 50-ka estimate for the main anoxia intensification during latest Rhaetian based on the contemporary uranium isotope record (*7*). Subsequently, the gradual falling limb of the δ^34^S_CAS_ excursion corresponds with the second phase of limited anoxia that extended into the Hettangian (*7*). The event also saw the intensification of the Panthalassan OMZ and the deposition of black shales on the Pangean margin and in the shelf seas of Europe. Shallowest water locations, such as MS, remained oxygenated. The coincidence of the δ^34^S_CAS_ excursion with the extinction losses implicates anoxia as an important factor in the crisis.

The late Permian and the Mesozoic Era were punctuated by recurring OAEs accompanied by hyperthermal events and enhanced weathering that coincide with the eruption of LIPs (*2*, *12*, *35*). Large positive S isotope shifts in seawater sulfate provide evidence of a greatly reduced marine sulfate reservoir and enhanced pyrite burial for many of these OAEs (*9*–*12*). We explain this generalized coincidence via a mechanistic linkage between low dissolved sulfate, enhanced sedimentary methane generation, and consequent elevated bottom-water O_2_ consumption. Hence, we propose that a low sulfate boundary condition before volcanically driven greenhouse warming events makes the expansion of anoxic conditions more likely and that associated feedbacks during the event extend the geographic reach and intensity of anoxia. Many of these events are preceded by increased evaporite burial fluxes, suggesting that this is the mechanism for sulfate removal from the ocean (*27*, *32*, *40*). Hence, the development of widespread anoxia during rapid warming may ultimately trace some of its origins to widespread rifting or other circumstances that create favorable conditions for evaporite deposition.

## MATERIALS AND METHODS

### CAS extraction and elemental analysis

Micritic limestone samples were targeted for the extraction of CAS, but a few shell fragments and sparitic samples were selected from the CP and BBR in the absence of pure micritic carbonate materials (see lithological description for individual sample in data file S1). For bulk limestone samples, weathered surface or crusts were removed before grinding to a fine powder using a TEMA laboratory agate disc mill. Shell fragments were powdered by hand using an agate mortar. We applied a modified and miniaturized CAS extraction method that follows the work of He *et al*. (*13*) and Newton *et al*. (*9*), ~10 g of powder of the bulk limestone or ~0.8 g of powder of shell fragments was first bleached in excess 6% NaOCl for 48 hours to oxidize organic sulfur and metastable sulfide minerals to soluble sulfate. The bleaching step was repeated for the pyrite-rich samples from the BBR. The bleached solution was retained after filtration through 0.2-μm polypropylene membrane syringe filters, acidified with 6 M HCl, followed by addition of saturated BaCl_2_, and left to precipitate BaSO_4_ over a week at ~2°C to decelerate the barite crystal growth rate. The solid bleached residue was then washed in 10% NaCl solution for 24 hours to remove easily soluble sulfate and other non-CAS sulfur-bearing compounds. This NaCl wash step was repeated five times to guarantee the removal of non-CAS water-soluble sulfur contaminates (*13*). No BaSO_4_ precipitate was seen upon addition of BaCl_2_ to the final wash filtrate. We also measured sulfur concentrations in these final wash filtrates using a Thermo Fisher iCAP 7400 radial inductively coupled plasma optical emission spectrometer (ICP-OES) in the Cohen Geochemistry Laboratory, University of Leeds, but no sulfur was detected. Note that NaCl washes are necessary after the NaOCl bleach step to ensure the removal of soluble sulfate contaminates that were generated during the bleaching process. The NaCl-washed solid residue was then treated with an excess 6 M HCl to extract CAS. The acid digestion was finished within 20 min to minimize the potential for oxidation of any remaining pyrite contaminates. The extracted CAS solution was retained by filtration through 0.2-μm polypropylene membrane syringe filters. An aliquot of the filtered solution was measured for the concentration of sulfur and other elements (Ca, Mg, Fe, Sr, and Mn) using the ICP-OES. The analytical precisions for these elements were better than 3%. Saturated BaCl_2_ was then added to the remaining filtered solution and left to precipitate BaSO_4_. The resulting BaSO_4_ precipitate was repeatedly washed by ultrapure water before being dried and weighed out for isotope measurement.

### S isotope measurement

Sulfur isotopic analysis of dried BaSO_4_ precipitate from both the bleached filtrate and CAS solution were also carried out in the Cohen Geochemistry Laboratory using an Elementar PYRO cube coupled to an IsoPrime continuous flow mass spectrometer. The sample was weighed into an 8 mm by 5 mm tin cup and combusted at 1150°C in a flow of helium (CP grade) and pure oxygen (N5.0). Complete combustion was obtained by passing the gas through tungstic oxide held at the same temperature. Excess oxygen was removed from the gas stream using pure copper wires held at 850°C, and water was removed using SICAPENT. The resulting SO_2_ gas was separated from any contaminating N_2_ or CO_2_ by temperature-controlled adsorption/desorption columns. All solid reagents were sourced from Elemental Microanalysis, UK, and all gases were from BOC, UK. The sample δ^34^S value is derived using the integrated mass 64 and 66 signals of the sample relative to those in a pulse of SO_2_ reference gas (N3.0). These values were calibrated to the international Vienna-Canyon Diablo Troilite (V-CDT) scale using a seawater-derived lab barium sulfate standard, SWS-3, which has been analyzed against the international standards NBS-127 (20.3 ‰), NBS-123 (17.01 ‰), IAEA S-1 (−0.30 ‰), and IAEA S-3 (−32.06 ‰) and assigned a value of 20.3‰, and an interlab chalcopyrite standard CP-1 assigned a value of −4.56 ‰. The precision obtained for repeat analysis of a laboratory check standard BaSO_4_ was ±0.3‰ (1 SD) or better.

### Rate method and the sulfur cycle box model

The rate method (*13*, *25*, *41*) was performed to estimate the maximum seawater sulfate concentrations using the parameters and calculation method described in the work of He *et al*. (*13*). The sulfur cycle box model follows the work of Witts *et al*. (*24*). Fluxes and parameters for the model are shown in table S1, and further information is given in the Supplementary Materials.

## SUPPLEMENTARY MATERIALS

Supplementary material for this article is available at http://advances.sciencemag.org/cgi/content/full/6/37/eabb6704/DC1
